# Thermal Modulation of Electrodeposition Stability in Sodium Metal Electrodes

**DOI:** 10.1002/advs.202515275

**Published:** 2025-10-13

**Authors:** Deep Chatterjee, Aditya Singla, Debanjali Chatterjee, Bairav S. Vishnugopi, Partha P. Mukherjee

**Affiliations:** ^1^ School of Mechanical Engineering Purdue University West Lafayette IN 47907 USA

**Keywords:** dendrite growth, electrodeposition stability, linear stability analysis, sodium metal electrode, soret effect, thermal modulation

## Abstract

Sodium metal batteries (SMBs) have gained interest due to the high natural abundance and lower cost of sodium (Na) compared to lithium (Li), making them a promising alternative to conventional Li‐based battery systems. However, a key challenge toward the commercial viability of SMBs lies in mitigating uneven electrodeposition and dendrite growth, stemming from inherent interfacial instabilities during Na plating. This work explores how electrodeposition stability in SMBs is governed by thermal conditions, which directly affect ionic transport and interfacial reaction kinetics. A range of thermal environments are explored using a phase‐field modeling (PFM) framework, with a particular emphasis on the influence of temperature gradient‐induced thermodiffusion (Soret effect) on deposition dynamics. A quantitative analysis of dendrite growth under varying thermal conditions is conducted to identify regimes that promote stable plating behavior. It is found that operational temperature serves as a strong modulator of plating instability by simultaneously influencing reaction kinetics and ion transport, and thermodiffusion under imposed temperature gradients further redistributes ionic flux and alters deposition morphology. This work provides new insights into the role of thermal landscapes in dictating interface evolution during Na metal plating and offers design guidelines for leveraging thermal conditions to enhance deposition stability in Na metal electrodes.

## Graphical abstract

Unstable electrodeposition in sodium metal electrodes during plating gives rise to dendritic morphologies. A comprehensive analysis of how various thermal profiles, including uniform and non‐uniform temperature distributions and thermodiffusion can affect electrodeposition instability has been performed. Mechanistic interface instability descriptors have been introduced to quantify dendritic growth.

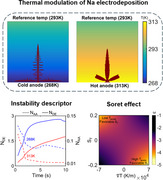

## 1 Introduction

The growing demand for electrified transportation, portable electronics, and grid‐scale systems necessitates the need for energy storage systems capable of delivering higher energy densities.^[^ ^1^, ^2^, ^3^ ^]^ As commercial batteries based on conventional porous anode architectures approach their theoretical energy density limits, metal anode‐based rechargeable batteries are emerging as a promising alternative for next‐generation energy storage systems. By replacing the intercalation‐type porous electrode entirely with a metal electrode, these systems eliminate the need for inactive host materials,^[^ ^4^ ^]^ thereby enabling a direct increase in energy density. In this context, existing research on metal anodes has primarily focused on lithium (Li) as the anode active material owing to its favorable electrochemical properties, such as a low reduction potential (−3.04 V compared to SHE) and high specific capacity (3860 mAh g^−1^).^[^ ^5^, ^6^, ^7^, ^8^ ^]^ However, alternate chemistries are now being considered in response to sustainability and resource availability concerns associated with Li.^[^ ^9^, ^10^, ^11^ ^]^ Among these, sodium metal batteries (SMBs), which utilize sodium (Na) as the anode active material, are gaining significant attention due to the earth abundance and broad geographical availability of Na.^[^ ^12^, ^13^ ^]^ In addition, the electrochemical potential of Na is sufficiently close to Li (−2.71 V compared to SHE), while also offering a competitive theoretical capacity (≈1166 mAh g^−1^).^[^ ^13^, ^14^ ^]^

Despite their promise, metal anode batteries, particularly SMBs, face significant challenges on the path to commercialization, including dendrite formation and low Coulombic efficiency.^[^ ^15^, ^16^, ^17^ ^]^ Additionally, the instability of the solid electrolyte interphase (SEI) leads to continuous consumption of active Na and electrolyte components.^[^ ^18^, ^19^ ^]^ This degradation process results in the formation of “dead Na” deposits‐ electrically isolated Na fragments that no longer participate in cycling and contribute to capacity loss.^[^ ^20^ ^]^ Such interfacial issues collectively undermine the electrochemical performance, with Na systems exhibiting a lower critical current density (CCD) threshold for safe operation relative to their Li counterparts.^[^ ^21^ ^]^ Thus, it becomes more important to understand and mitigate the inherent interfacial instabilities in SMBs.

To investigate dendrite formation in alkali metal anodes, extensive experimental characterization has been performed, offering insights into deposition dynamics. While the nucleation and growth mechanisms are well established for Li metal anodes,^[^ ^22^, ^23^ ^]^ research has been relatively sparse for Na metal systems. Existing studies on Na dendrite formation have revealed several key distinctions from Li. Na has a larger atomic radius than Li. As a consequence, Na exhibits a larger molar volume (2.4 ×10^−5^ m^3^ mol^−1^), as compared to Li (1.3 ×10^−5^ m^3^ mol^−1^).^[^ ^15^, ^24^ ^]^ This difference manifests in the form of increased volume of electrodeposit for Na compared to Li for an equivalent plated charge capacity. In addition, Na metal has a lower surface energy than Li, which has been shown through DFT calculations.^[^ ^25^, ^26^, ^27^ ^]^ This makes the formation of fresh interfaces thermodynamically more favorable for Na electrodeposits versus Li. Morphologically, this manifests as dendritic, less compact Na electrodeposits than Li.^[^ ^15^ ^]^ In addition, Na has a lower hardness compared to Li, which makes the Na dendrites “soft” and more prone to mechanical stress.^[^ ^28^ ^]^ Also, unlike Li, Na has a greater tendency to form electrically isolated “dead” fragments through base dissolution of deposits, which results in reduced Coulombic efficiency.^[^ ^29^ ^]^ Direct experimental studies on Na metal anodes with different electrolyte systems have also highlighted the role of the solvation structure in dictating Na deposition stability.^[^ ^30^ ^]^

The physical origin of unstable interfacial deposition and the underlying mechanisms driving dendrite growth in metal anodes have been extensively examined in past studies. An early theoretical framework by Bazant et al.^[^ ^31^ ^]^ generalized reaction kinetics by incorporating thermodynamic driving forces. Thermodynamic understanding of dendrite initiation has elucidated the role of key parameters, such as temperature, in influencing dendrite initiation and growth by modulating ionic transport and reaction rates.^[^ ^32^ ^]^ Ely et al.^[^ ^33^, ^34^ ^]^ introduced the concept of thermodynamic and kinetic critical radii, establishing nucleation size thresholds that dictate whether a nucleus will grow or shrink upon further plating. Dendrite initiation has been linked to ionic diffusion limitation near the electrode surface, and the onset time for such instabilities is dictated by the Sand's time of the system.^[^ ^35^ ^]^ However, deviations from the classical Sand's time behavior have been observed, particularly for high applied current densities or reduced inter‐electrode spacing, as shown by Mistry et al.^[^ ^36^ ^]^ They also introduced the concept of “confined electrolyte”, which has been adopted in the present work to understand deposition instability. Building on the physical understanding of interface evolution, several studies have also attempted to model dendritic growth during electrodeposition.^[^ ^37^, ^38^, ^39^, ^40^, ^41^, ^42^, ^43^, ^44^, ^45^, ^46^, ^47^ ^]^

Operational temperature plays a critical role in governing interfacial instability by modulating both ionic transport and the kinetics of electrodeposition. Experimentally, it has been shown that temperatures up to 60°C can promote compact Li deposition through improved ion transport.^[^ ^45^ ^]^ Concurrently, in situ microscopy has further demonstrated that high salt concentration and elevated temperature improve deposition stability during plating.^[^ ^48^ ^]^ Experimental study by Matsumoto et al.^[^ ^49^ ^]^ studied Na metal deposition morphology variation with temperature in liquid electrolyte and have observed a transition from thin, whisker‐like Na morphology at low temperatures (273–293K), to a deposition morphology that becomes smoother and more compact at high temperature (≈363K). Further, the effect of an applied thermal gradient has also been experimentally studied for lithium‐ion cells,^[^ ^50^ ^]^ where it was found that keeping a cold anode and warmer cathode led to faster battery degradation as opposed to the condition where the anode is warm, and the cathode is kept at a lower temperature. Experimental studies have also been conducted for SMBs at very low temperatures, which have focused on operational challenges due to sluggish kinetics and proposed carboxylate^[^ ^51^ ^]^ or carbonate^[^ ^52^ ^]^ based electrolytes to obtain stable cycling under these conditions. Modeling studies also complement this strong coupling between thermal conditions and interface instability, offering mechanistic insights into the role of temperature in regulating deposition morphology.^[^ ^40^, ^43^, ^47^ ^]^ However, the majority of prior studies in this context have focused on Li as the active material.^[^ ^40^, ^43^, ^47^, ^48^, ^53^, ^54^ ^]^ In addition, thermodiffusion (Soret effect),^[^ ^55^, ^56^, ^57^ ^]^ which drives ion migration under thermal gradients and may significantly influence interfacial dynamics, has been largely overlooked in battery studies. The potentially significant effects of the thermal operating condition during plating, as observed experimentally, necessitates an understanding of the fundamental mechanisms at play that govern the dendritic growth process and their modulation under different thermal conditions in order to mitigate unstable dendritic growth.

In this work, we investigate the electrodeposition instability of Na metal electrode in liquid electrolyte under distinct thermal landscapes, including uniform temperature, imposed temperature differentials, and spatial temperature gradients using a phase field modeling framework. We consider the applied thermal distribution to be invariant with time, that is, we do not consider the effects of internal heat generation^[^ ^40^ ^]^ (due to ohmic heat generation or kinetic heating during plating) in order to specifically decouple the role of temperature in dictating anode instability during the electrodeposition process. We conduct a relative quantitative analysis to assess deposition instability across these conditions. Our findings reveal that temperature, particularly at the anode, plays a decisive role in governing interfacial stability. We show that elevated temperatures mitigate growth instabilities, while thermodiffusion further modulates deposition behavior in the presence of temperature gradients. To complement the numerical phase field results, we employ linear stability analysis (LSA) to assess electrodeposition instability,^[^ ^58^, ^59^, ^60^ ^]^ and consistent trends were observed across both approaches. Overall, this study provides a comprehensive perspective on the role of thermal conditions in Na metal deposition and delineates the regimes that favor stable Na plating.

## 2 Results and Discussion

**Figure 1:**
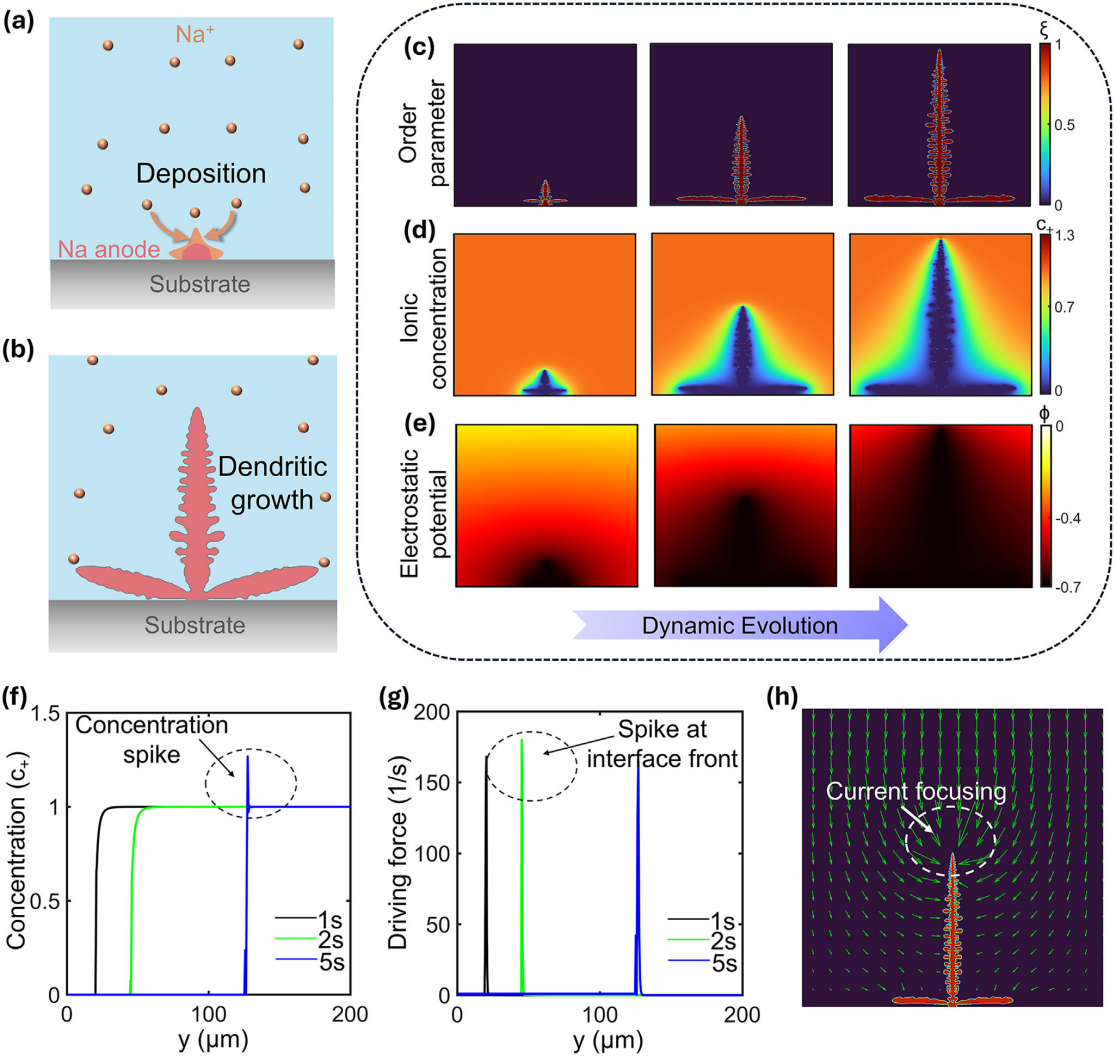
a,b) Schematic illustration of the electrodeposition process leading to branched, fractal‐like growth from an initial Na deposit. c–e) Time evolution of coupled variables: order parameter, non‐dimensional ionic concentration, and electrostatic potential, respectively. f,g) Time evolution of ionic concentration and driving force, respectively at x = 100 µm. h) Electric field showing current focusing near the dendrite tip.

A description of the model employed in this work, along with representative results, is shown in **Figure** **1**. The initial domain consists of a nucleated Na anode surrounded by liquid electrolyte, with a fixed negative plating potential applied at the anode (Figure 1a). During plating, the initial nucleation on a substrate is followed by further deposition on it, leading to dendritic growth (Figure 1b). Electrodeposition is governed by the time evolution of coupled variables: the order parameter (representing the non‐dimensional concentration of Na metal), the non‐dimensional ionic concentration of Na^+^, and the electrostatic potential; the constitutive governing equations associated with these variables are stated in Section S1 (Supporting Information), and the relevant model parameters are listed in Table S1 (Supporting Information). These variables collectively represent the state of the system at each time instant, and their transient evolution is represented in Figure 1c–e. Herein, the order parameter, representing the morphology of the Na deposit, evolves in a branched, fractal‐like manner, as shown in Figure 1c. Concurrently, in Figure 1d, the ionic concentration of Na^+^ is shown to evolve in a more diffuse manner, exhibiting a gradual decrease in cation availability toward the advancing anode front. A region of low ionic concentration near the electrodeposit (indicated by the blue region) thus results from the local ionic consumption as Na^+^ ions reduce to Na metal at the reaction sites. Figure 1e shows the electrostatic potential distribution in the domain, with the region surrounding the deposit marked by a large, negative value of electrostatic potential due to the high electronic conductivity of Na. At later stages of dendritic growth, increased potential and concentration gradients drive ionic flux toward the dendritic tip, resulting in Na^+^ crowding and a local ionic concentration above the bulk value. This is illustrated quantitatively through plots of ionic concentration at x = 100 µm (vertical midline) in Figure 1f. In the initial stages, the nondimensional ionic concentration increases from 0 to 1 along the growth direction, corresponding to the metal deposit and electrolyte phases, respectively. The sharp transition observed in these profiles marks the interfacial region and correlates with the location of the dendrite tip. As plating progresses, this transition point shifts forward with time, indicating dendrite growth. At later stages of dendritic growth, there is an intermediate spike in concentration above 1 (bulk value) near the front due to crowding of Na^+^ ions.^[^ ^39^ ^]^ The electrochemical driving force also shows a localized spike near the dendrite tip in a similar plot and remains negligible elsewhere, as shown at x = 100 µm in Figure 1g. This further demonstrates that electrodeposition is locally confined to the interface, the location of which corresponds to the sharp spikes observed. Finally, Figure 1h shows the distribution of the electric field across the domain, showing current focusing near the dendrite tip, which serves as the principal site for interfacial kinetics. This focusing of the electric field is a key contributor in driving the Na^+^ ions toward dendritic tips, as discussed before.

In the subsequent sections, we explore in detail the effects of various thermal operating conditions on electrodeposit growth morphology during Na plating.

### 2.1 Uniform Temperature Distribution

**Figure 2:**
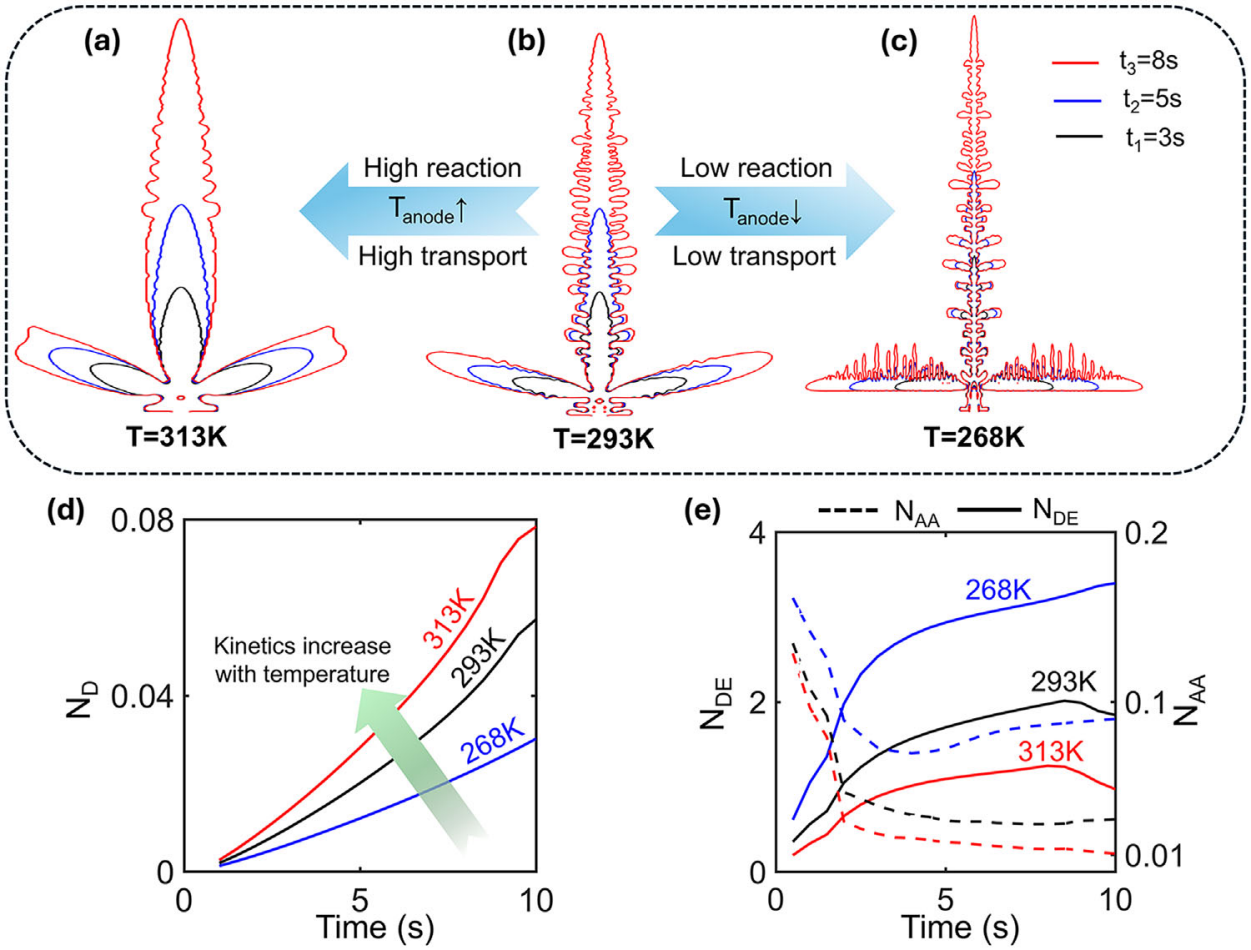
Evolution of dendrite morphology at different uniform temperatures: a) 313K, b) 293K, and c) 268K. d) Temporal evolution of N_D_; N_D_ increases with time at different uniform temperatures, showing an increase in reaction kinetics with temperature. e) Variation of N_AA_ and N_DE_ with time for different domain temperatures, showing a higher instability (higher values of the corresponding metrics) at lower uniform temperatures.

Temperature serves as a key modulator of dendritic morphology in electrodeposition, impacting both reaction kinetics and ion transport via an Arrhenius correlation. The sensitivity of these processes to temperature variation is dependent on the activation energy of the corresponding process (Table S1, Supporting Information). **Figure** **2**a–c shows the time evolution of the electrodeposit profile at the interface for three different uniform temperature conditions: T = 268K (low temperature), 293K (reference temperature), and 313K (high temperature), respectively. An increase in temperature above the reference temperature leads to more electrodeposition and a less branched structure (Figure 2a). This behavior can be attributed to increased reaction rates, enhanced ion transport, and greater ion availability near the anode front at elevated temperatures. In contrast, at low temperatures, thinner and more branched dendritic growth is observed, resulting from a lower ion availability at the interface and reduced electrodeposition rate (Figure 2c).

To quantify the dendrite growth instability, we introduce metrics that have been used subsequently throughout the study and are represented schematically in (Figure S3 and Section S3, Supporting Information). Since the diffuse interface serves as the seat for reaction, the total interfacial area correlates with the rate of electrodeposition of Na^+^. Additionally, cations must be present at the reaction sites for deposition. Thus, the dual condition of the presence of an interface and a finite cation concentration in that region helps define the effective active area for reaction. The ratio of the effective active area to the total amount of metal deposited is denoted in this work as the normalized active area (N_AA_). This indicates how much “fresh interface” is created per unit volume (area in 2D) of metal deposited. A less branched growth morphology would lead to a relatively lower N_AA_ value (implying a relatively stable growth), while a more branched dendritic structure denotes a higher N_AA_ value and unstable growth.

(1) $$\begin{array}{c} \;N_{\mathrm{AA}}=\frac{\text{Effective}\;\text{active}\;\text{area}}{\text{Total}\;\text{deposit}\;\text{area}} \end{array}$$

In correlation with N_AA,_ a second metric of instability, the normalized depleted electrolyte (N_DE_), is defined. As evident from the ionic concentration profiles, a zone of low ionic concentration develops adjacent to the electrodeposit. By selecting a suitable threshold value of “low ionic concentration”, the depleted region can be quantified. This is subsequently normalized by the total deposit quantity, as before. It is subsequently shown that N_DE_ is correlated with N_AA_, with higher values corresponding to more branched dendritic growth and lower values associated with more compact morphologies. Moreover, it is shown in (Section S3 and Figure S4, Supporting Information) that N_DE_ is a more consistent measure of instability, compared to N_AA_, making it a more reliable metric.

(2) $$\begin{array}{c} \;N_{\mathrm{DE}}=\frac{\text{Depleted}\;\text{electrolyte}}{\text{Total}\;\text{deposit}\;\text{area}} \end{array}$$

Furthermore, while representing the quantity of Na deposited in Figure 2d and thereafter, it has been scaled by the total area of the domain and termed as N_D_.

(3) $$\begin{array}{c} \;N_{D}=\frac{\text{Total}\;\text{deposit}\;\text{area}}{\text{Domain}\;\text{area}} \end{array}$$

The increase in reaction kinetics with temperature is shown in Figure 2d, as N_D_ increases with temperature. Figure 2e describes the quantitative variation of N_AA_ and N_DE_ with time at different spatially uniform domain temperatures. A lower uniform temperature (268K) causes more dendritic (and hence more unstable) growth, whereas a higher temperature (313K) results in less branching and a smoother, more stable deposition. This trend is quantitatively reflected in this figure, where both N_AA_ and N_DE_ decrease with increasing uniform domain temperature.

### 2.2 Non‐Uniform Temperature Distribution

**Figure 3:**
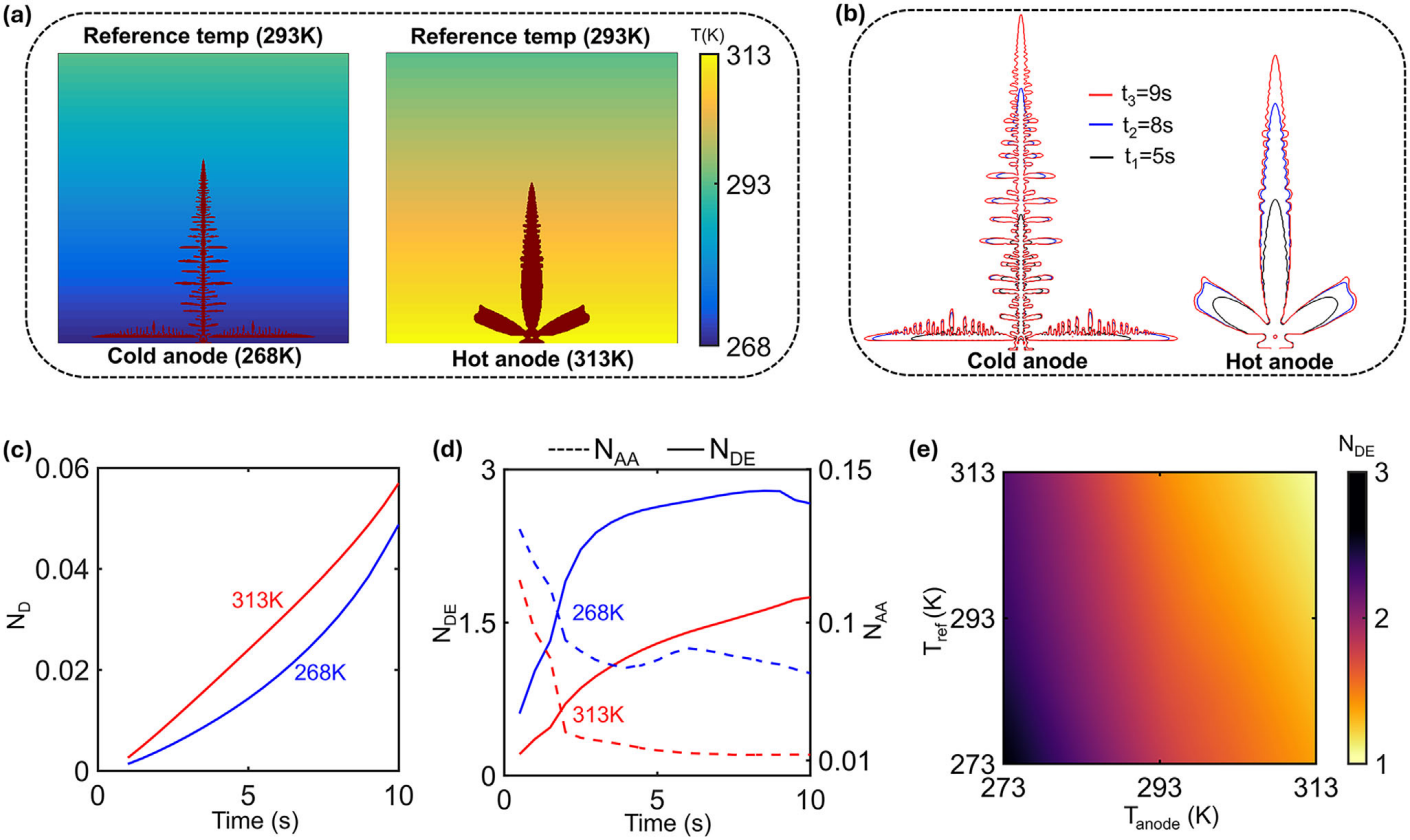
a) Unstable growth for low anode temperature (286K) and smoother deposition for high anode temperature (313 K). b) Time evolution of dendritic growth for a cold and a hot anode. c) Variation of N_D_ with time for low and high anode temperatures. d) Variation of N_AA_ and N_DE_ with time for different anode temperatures, showing improved stability with increasing anode temperature. e) Phase map showing the variation of N_DE_ over a range of T_anode_ and T_ref_.

To delineate the role of spatially varying thermal conditions on deposition instability, we consider the simple case of a linear temperature variation across the domain, with the anode kept either hot (313K) or cold (268K), and a fixed reference temperature (293K) at the other end. Further, the temperature profile is assumed to stay constant with time, that is, the effects of internal heat generation on temperature distribution are not considered in this study. The deposition profile at a representative time, overlayed with the temperature profile for the two conditions, is shown in **Figure** **3**a. It is seen that higher anode temperatures lead to less branched (or more stable) deposition compared to a lower anode temperature. The time evolution of the dendrite profiles is shown in Figure 3b for the two cases. Using the same metrics as defined in the previous section, the total normalized deposition (N_D_) and the relative stability of the dendritic growth (N_AA_ and N_DE_) are analyzed in Figure 3c,d, respectively. It is seen that higher anode temperatures lead to increased net deposition (Figure 3c) due to a higher rate of electrodeposition. Figure 3d compares electrochemical stability across the three different anode temperatures, showing improved stability (lower N_AA_ and N_DE_) as the anode temperature rises. The net deposition trend in Figure 3c reveals that deposition progresses nearly linearly with time at higher temperatures, whereas at lower anode temperatures, an exponential increase in deposition is observed. This behavior is attributed to higher branching at low anode temperatures, which increases the interfacial area and thus creates more active sites for electrodeposition, initiating a cascade effect. So far, it has been shown that lower uniform temperatures and lower anode temperatures lead to more branched deposition. To confirm the relative importance of the anode temperature and reference temperature in dictating this observed growth instability, the variation of N_DE_ with reference temperatures (T_ref_) and anode temperature (T_anode_) is presented in Figure 3e. It is revealed that T_anode_ primarily dictates the growth instability, with higher anode temperatures resulting in lower N_DE_ (improved relative stability) and vice versa. In contrast, there is negligible variation of N_DE_ as T_ref_ increases, with only a slight improvement in deposition stability (slightly lower N_DE_ values corresponding to an increase in T_ref)._

### 2.3 Soret Effect

Thermodiffusion (or the Soret effect) refers to species diffusion driven by thermal gradients in the system. In this study, we consider how solvated Na^+^ ions may be affected by induced thermal gradients in the domain. The magnitude of the Soret effect is dictated by the value of the Soret coefficient (S_T_), and it manifests in the form of a concentration flux in the domain induced by existing thermal gradients (= D_T_ c∇T) where c is the solvated Na^+^ concentration and D_T_ represents the thermodiffusion coefficient.^[^ ^61^ ^]^ The directionality of thermodiffusion depends on the sign of S_T_, which may change in the same system depending on factors such as temperature and composition of the species. A positive value of S_T_ (S_T_>0) leads to a propensity of ions being driven to low temperature zones (showing thermophobic behavior), while a negative value of S_T_ (S_T_<0) drives ions toward high temperature regions (showing thermophilic behavior). Past research on the Soret effect in dissolved salts shows this phenomenon of paradigm shift from thermophilic to thermophobic behavior and vice‐versa as the salt concentration or temperature changes.^[^ ^62^, ^63^ ^]^ Further details of the modified governing equations and the methodology adopted in the study accounting for thermodiffusion effects are provided in Section S1 (Supporting Information).

**Figure 4:**
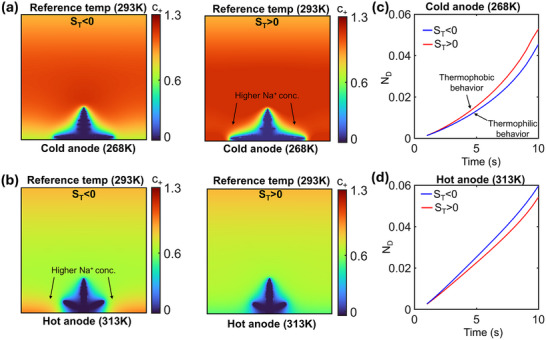
Nondimensional ionic concentration profile under Soret effect for a) cold anode (268 K), and b) hot anode (313 K), showing greater concentration near the anode for conditions where Soret effect drives Na^+^ toward the anode. c,d) Variation of N_D_ with time for the two cases, showing larger deposition for conditions under which Na^+^ ions are driven toward the anode. For all cases, the reference temperature at the opposite boundary is fixed at 293K.

The consequences of the Soret effect (thermodiffusion) are shown in **Figure** **4** under a linear temperature variation across the domain, as before. Figure 4a,b shows the ionic concentration profile for low anode temperature (268K) and high anode temperature (313K), respectively, for both negative and positive S_T_, accounting for possible thermophilic and thermophobic behavior of the solvated Na^+^ ions. There is a higher Na^+^ concentration near the anode in Figure 4a for positive S_T_ since the anode is kept at a lower temperature, and ions crowd near the low temperature zone for positive S_T_. In contrast, as shown for the negative value of S_T_, thermodiffusion acts in the opposite direction, driving cations away from the anode. This effect has a direct consequence on the net deposition‐ a higher deposition is observed for S_T_>0 when T_anode_ is low (Figure 4c).

Figure 4b shows ionic concentration profiles for high anode temperature (313K), also accounting for both thermophobic and thermophilic behavior. Here, a comparison reveals an opposite trend to earlier observations: S_T_<0 results in a higher ionic concentration near the anode, since ions show thermophilic behavior under this condition when the anode is at a higher temperature, whereas the reverse trend is observed for S_T_>0. The corresponding variation in N_D_ (Figure 4d) confirms the behavior, showing a greater deposition for S_T_<0.

**Figure 5:**
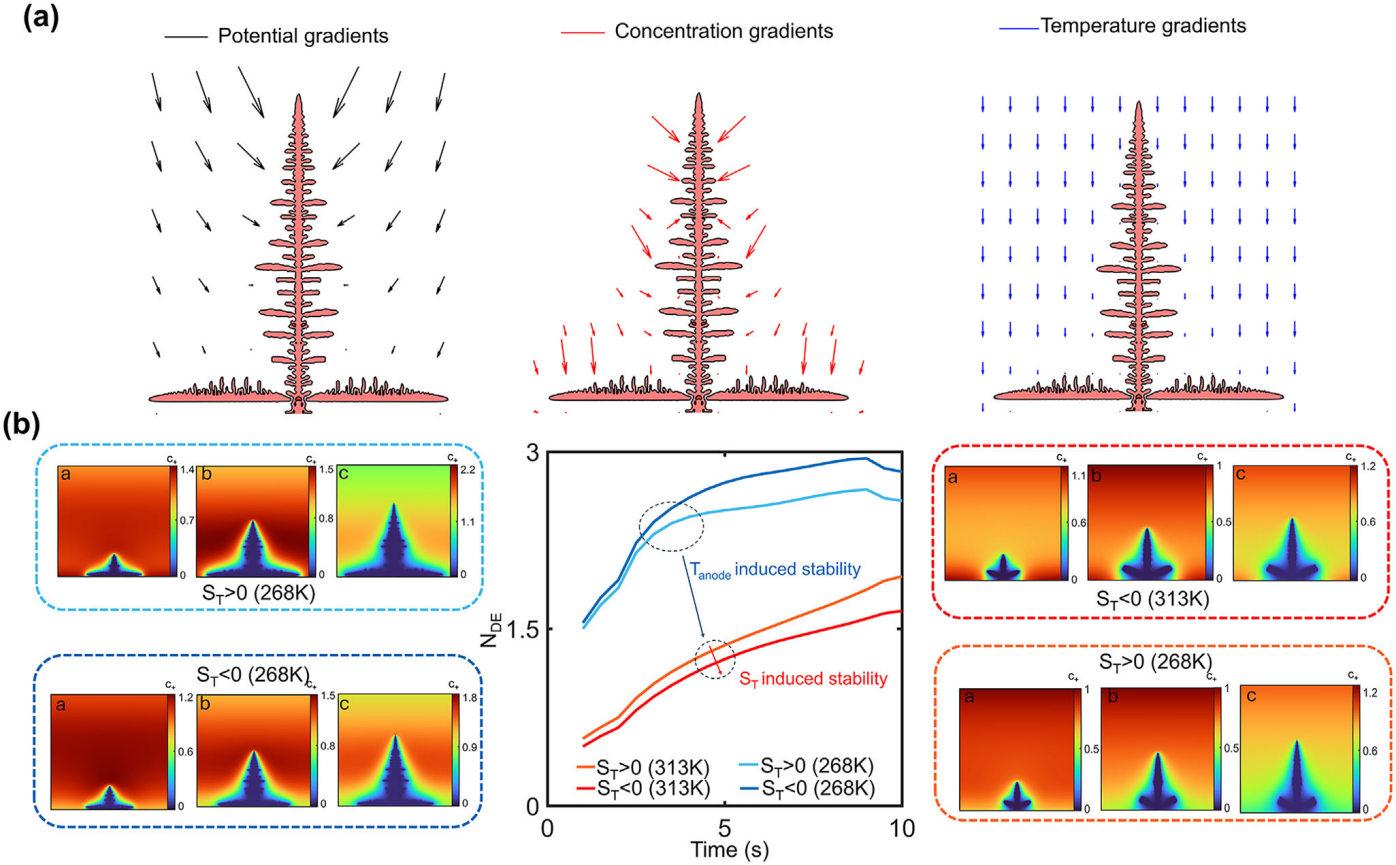
a) Potential, concentration, and temperature‐driven ionic flux directions for low T_anode_ and S_T_>0 condition, with the size of arrows representing their relative magnitude. b) Variation of N_DE_ with anode temperature and S_T_, showing that thermodiffusion conditions driving cation toward the anode lead to more stability; flank plots showing Na^+^ concentration at different times under different thermodiffusion and anode temperature conditions.

To further study the Soret effect and explain the trends observed, **Figure** **5**a shows the directionality of the individual driving forces in the Nernst Planck equation, that is, potential gradients, concentration gradients, and thermal gradients for a low T_anode_ and a positive S_T_. Here, the arrow sizes correspond to the relative magnitude of the different driving agents. Ionic flux due to potential gradients generally exhibits the highest magnitude, except near the dendrite tips, where fluxes due to concentration gradients are also significant. Moreover, the concentration gradient‐driven ionic flux is localized around the dendrite, as significant concentration gradients exist only in that region. When comparing the direction of these separate fluxes, it may be observed that potential gradients, and to a good extent, the concentration gradients primarily drive ions toward the tip of the dendritic branches due to their higher magnitude in these regions. In contrast, thermal gradient‐induced ionic flux is directed vertically downwards following the linear temperature gradient under the condition of a low anode temperature with S_T_>0. As a result, Na^+^ availability is improved near the base of the electrodeposit, leading to increased deposition in the basal region rather than exclusively at the dendrite tips.

Figure 5b primarily compares deposition stability by plotting N_DE_ versus time (central plot) under the conditions explored in Figure 4. Two distinct groups are formed for different values of T_anode_, with high T_anode_ corresponding to the cluster at the bottom showing lower N_DE_ values (light and dark red curves) and low T_anode_ corresponding to higher N_DE_ values (light and dark blue curves, corresponding to the cluster at the top). Further demarcation in the N_DE_ trend is produced by a difference in the S_T_ values. It is seen that the thermodiffusion conditions that promote ionic deposition toward the anode base also promote deposition stability with a lower N_DE_ than their counterparts. Thus, for low anode temperature, S_T_>0 (light blue curve) and for high anode temperatures, S_T_<0 (dark red curve) lead to more stable deposition behavior. Further, the distinction in N_DE_ values due to S_T_ is more prominent for low anode temperatures, showing a larger gap with changing S_T_ values than at high anode temperatures, where the curves are closer. This is because the effect of thermodiffusion depends only on the magnitude of thermal gradients and the value of S_T_. Thus, the relative contribution of thermodiffusion is high when the intrinsic temperature‐induced deposition stability is comparatively less, such as for low T_anode_ (while the thermal gradient and S_T_ magnitude stay the same), which leads to the aforementioned observation. Contours of non‐dimensional Na^+^ concentration have also been shown at different time instants for the different conditions explored for better clarity.

### 2.4 Linear Stability Analysis (LSA)

LSA is a useful tool to analyze the effect of applied surface perturbations. Using LSA, we obtain an instability metric, the growth rate (ω), for different perturbed surfaces, characterized by wavenumber values (k). In this context, a lower ω value would signify a lower rate of perturbation growth, implying relative stability. Details of governing equations for LSA analysis employed in this study have been provided in Section S2 (Supporting Information). For a preliminary analysis, line plots of perturbation growth ω have been plotted against k in (Section S3 and Figure S5, Supporting Information), corresponding to uniform and differential temperature conditions explored in Section 2.1 and 2.2, respectively. This aligns with the PFM results, exhibiting higher ω values for low uniform temperature and low anode temperature conditions. These observations establish a consistent correspondence between the outcomes of the PFM and LSA frameworks. In the following section, LSA is employed to investigate the influence of thermodiffusion on deposition stability.

**Figure 6:**
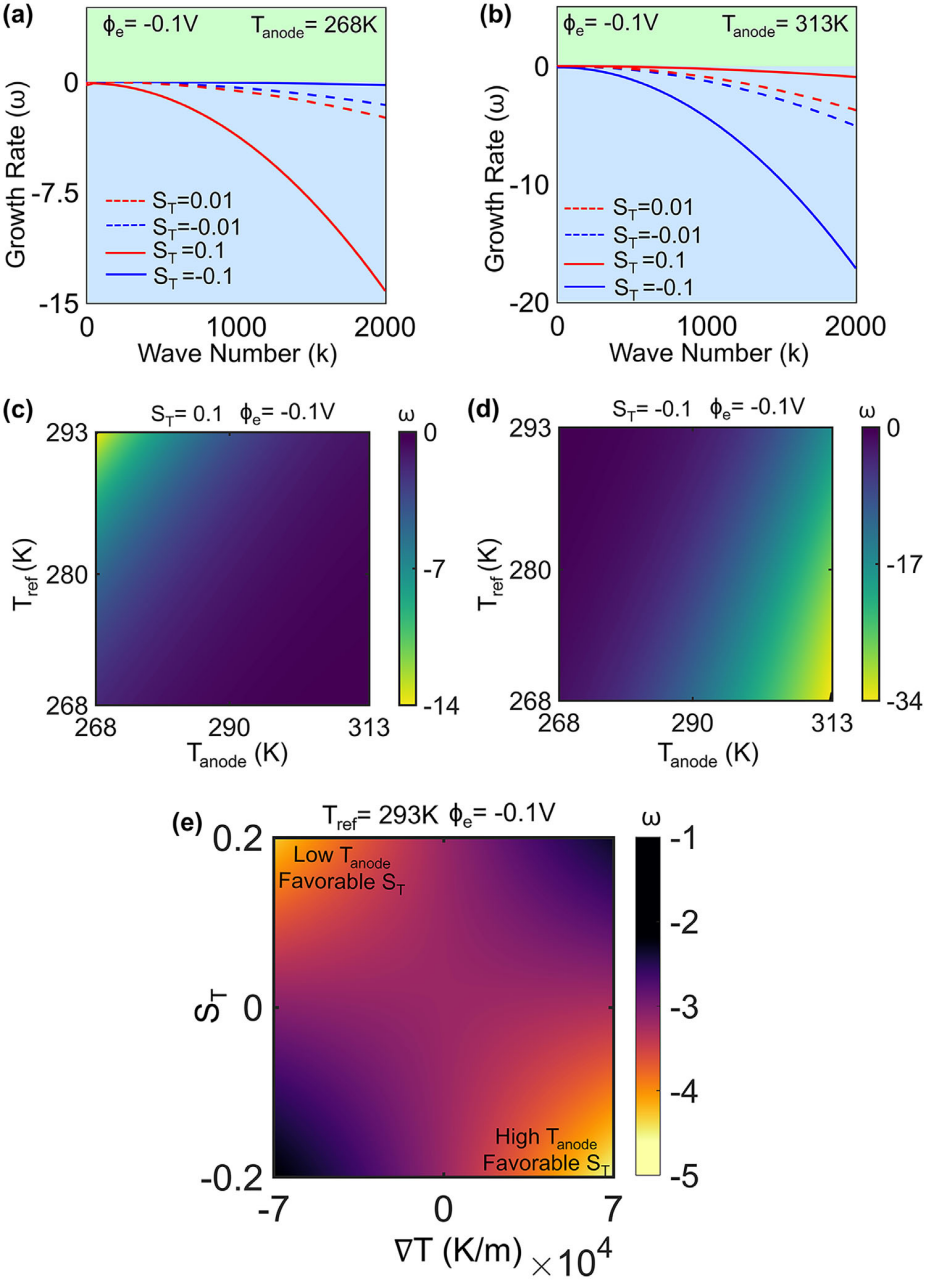
a,b) LSA line plots for different values of T_anode_ and S_T_. c,d) Phase maps using LSA showing stability regimes under varied T_anode_ and T_ref_ for S_T_ = 0.1 and S_T_ = −0.1, respectively. e) S_T_ vs ∇T phase map exhibiting stable and unstable deposition zones.

LSA plots under comparable conditions as in PFM (with the inclusion of the Soret effect) have been shown in **Figure** **6**. Line plots of ω versus k variation are shown in Figure 6a,b for different anode temperatures. The observed trends confirm the results from PFM, showing that for low T_anode_, S_T_>0, and for high T_anode_, S_T_<0 is more conducive to stable deposition, showing relatively lower ω under these conditions. The effect of varying the magnitude of S_T_ has also been shown, revealing that the stable/unstable growth behavior amplifies when the magnitude of S_T_ increases.

Further, phase maps using LSA plot ω at k = 2000 over a range of T_anode_ and T_ref_ for S_T_ = 0.1 and −0.1, respectively (Figure 6c,d). These results also align with previous conclusions from PFM regarding thermodiffusion‐induced stability. For S_T_>0, lower T_anode_ promotes thermodiffusion‐induced stability, while the stability also improves under these conditions as the disparity between T_anode_ and T_ref_ increases, since it leads to larger thermal gradients in the domain. Hence, the highest stability is observed in the top‐left corner of the phase map for S_T_>0, corresponding to low T_anode_ and high T_ref_. A reverse trend is observed when the S_T_ sign is flipped, since thermodiffusion now acts in the opposite direction.

A comprehensive phase map showing the interplay between S_T_ and temperature gradient (∇T = $\frac{T_{anode}-T_{ref}}{L})$ is shown in Figure 6e. Temperature gradient in this context is altered by changing T_anode_ while keeping a fixed T_ref_ and domain length. There is a distinct demarcation in stability that results from the value of S_T_, with relatively stable growth (low ω zones) for positive S_T_ and negative ∇T (T_anode_< 293K), where Na^+^ is driven toward the anode, and vice versa. Thus, the bottom right and top left regions correspond to higher stability, and lower values of ω are observed in these zones, while a relatively unstable growth is observed near the other two corners of the phase map.

## 3 Conclusion

This study analyzes the electrodeposition stability of Na metal anodes under different thermal landscapes during plating. We systematically investigate the role of thermal operating conditions, including uniform domain temperature, linear temperature gradients between fixed anode and reference temperatures, and thermodiffusion (Soret effect), in determining the growth morphology of a nucleated Na electrodeposit. Elevated domain temperatures enhance both reaction kinetics (destabilizing effect) and ion transport (stabilizing effect), with these competing influences determining the net stability. In order to specifically delineate the role of temperature in governing plating instability, internal heat generation has not been considered in this work. In the presence of ohmic and kinetic heating, high heat generation near the electrodeposit interface is expected to increase the local temperature in this region, especially at high plating current densities‐ thereby modulating ionic transport and even altering local temperature gradients near the interface, making it challenging to decouple the specific role of temperature induced instability. In this study, it was shown that the anode temperature emerges as a dominant factor in governing deposition behavior: lower anode temperatures promote unstable, dendritic growth, whereas higher anode temperatures yield smoother, more uniform deposits. This study introduced unique metrics in the form of N_AA_ and N_DE_ to reliably quantify such instabilities, with a higher value of these normalized metrics corresponding to dendritic, branched morphology and a lower value to smother, more compact deposits. Under a linear temperature gradient, the Soret effect modulates electrodeposition morphology by altering ion migration. We find that when thermodiffusion drives ions toward the anode base, deposition stability improves, with ion enrichment at the deposit base suppressing the formation of new reactive interfaces and promoting basal growth. Further, using LSA, we complement the results derived from the PFM and demonstrate a correspondence between the two methods for all the thermal landscapes considered in the study. Overall, this study underscores the critical role of thermal management in controlling electrodeposition stability, emphasizing strategic temperature regulation and careful electrolyte selection as ways to mitigate instabilities. Specifically, maintaining higher temperature near the anode can significantly enhance stability by improving localized ion transport, whereas exploiting favorable thermodiffusion (via tailored temperature gradients and employing electrolytes with high intrinsic S_T_) can further optimize Na deposition morphology. It should further be noted that an increase in battery temperature can also simultaneously promote unwanted side reactions or electrolyte decomposition,^[^ ^64^ ^]^ which can be detrimental and may also culminate in thermal runaway.^[^ ^21^ ^]^ Thus, methods to mitigate dendrite formation that selectively increase the temperature near the anode, such as the application of pulse current^[^ ^53^, ^54^ ^]^ emerge as practical pathways for stable battery operation, as this promotes localized heating near the anode (improving local transport properties), while preventing a sustained rise in temperature.

## Conflict of Interest

The authors declare no conflict of interest.

## Supporting information



Supporting Information

## Data Availability

The data that support the findings of this study are available from the corresponding author upon reasonable request.
