# Supplementary material for: Thermal Modulation of Electrodeposition Stability in Sodium Metal Electrodes
Source: Adv Sci (Weinh). 2025 Oct 13;13(2):e15275. doi: 10.1002/advs.202515275 (PMC12786378; doi:10.1002/advs.202515275)
Supplement: Supplementary file 1 — Supporting Information [file ADVS-13-e15275-s001.docx]

**Supporting Information**

**Thermal Modulation of Electrodeposition Stability in Sodium Metal Electrodes**

*Deep Chatterjee, Aditya Singla, Debanjali Chatterjee, Bairav S. Vishnugopi and Partha P. Mukherjee**

School of Mechanical Engineering, Purdue University, West Lafayette, IN 47907, USA

^*^*Correspondence*: pmukherjee@purdue.edu

1. **Phase Field Model**

Phase Field Model (PFM) solves coupled transient PDEs involving the non-conserved order parameter ξ (representing the non-dimensional concentration of Na in the deposit phase), non-dimensional ionic concentration c_+_ (w.r.t the bulk Na^+^ concentration $c^{0}$) and the electrostatic potential ϕ. The non-dimensional governing equations^1^ are defined below:

$$\begin{aligned} \frac{\partial\xi}{\partial t} = -L_{\sigma} [g'(\xi) - \kappa\nabla^{2}\xi] - L_{\eta} h'(\xi) [exp(\frac{\left( 1-\alpha\right)nF\eta_{\alpha}}{RT}) - c₊ exp(\frac{-\alpha nF\eta_{\alpha}}{RT})]\#\left( 1 \right) \end{aligned}$$

$$\begin{aligned} \frac{\partial c₊}{\partial t}= \nabla\cdot[D_{eff} (\nabla c₊ + \frac{nFc₊\nabla\phi}{RT})] - \frac{cₛ}{c^{0}}\frac{\partial\xi}{\partial t} \#\left( 2 \right) \end{aligned}$$

$$\begin{aligned} \nabla\cdot(\sigma_{eff} \nabla\phi) = nFcₛ\frac{\partial\xi}{\partial t}\#\left( 3 \right) \end{aligned}$$

Here, $L_{\sigma}=\frac{i_{0}\gamma}{RT nF \kappa{C_{s}}^{2}}\left( \alpha c_{+}^{\alpha}e^{\frac{-\alpha nF\phi}{RT}}+\left( 1-\alpha\right)c_{+}^{-(1-\alpha)}e^{\frac{\left( 1-\alpha\right)nF\phi}{RT}} \right)$ is the interfacial mobility parameter and $L_{\eta}=\frac{i_{0}\gamma}{nF \kappa C_{s}}$ is the kinetic parameter.^2^ Equation (1) arises from taking the variational derivative of the free energy in the system and relating that to the deposition growth using the Allen Cahn model. Here $g\left( \xi\right)$is defined as $W\xi^{2} \left( 1-\xi\right)^{2}$, representing a double well function (with barrier height W/16) which translates to the bulk free energy (non-zero only in the diffuse interface). The anisotropy coefficient is defined as $k=k_{0}\left( 1+\delta cos\left( \omegaϴ \right) \right)$, $ϴ$ representing the orientation from the crystallographic axis, $\delta$ representing the anisotropy strength and $\omega$ representing the mode of anisotropy (= 4 for BCC crystal structures). Although, we use the coupled governing Equations (1-3) to study Na metal electrodeposition, it may be noted that this Allen Cahn type PFM is a general model and can reliably be applied to study the electrodeposition of other metals^3^ in liquid electrolyte systems. Further, in this study, in order to accurately reflect the effects of Na metal evolution, we have used appropriate expressions for the interfacial mobility and kinetic parameter as described before, derived from fundamental electrodeposition equations.^1^ Time evolution of order parameter in Equation (1) can be split into two components: the first term modulates the interface with two competing effects between the thickness of the diffuse interface (corresponding to bulk free energy of the double well function) and the sharpness or the gradient of the order parameter (corresponding to the gradient free energy). The second term introduces the kinetics of deposition (with $\eta_{a}$ representing the activation overpotential) which is confined only to the interface using the derivative of a suitable interpolating function $h^{'}\left( \xi\right)$. This term represents the electrochemical driving force. The choice of interpolating function must satisfy $h\left( 0 \right)=0$, $h\left( 1 \right)=1$ and $h^{'}\left( 0 \right)=h^{'}\left( 1 \right)=0$. Since we have conducted this study considering liquid electrolytes, mechanical stress induced effects such as self-diffusion in the solid phase or creep deformation of the dendrites have not been considered in the governing equation for the order parameter evolution. The unique properties of Na metal, compared to the more conventionally studied Li deposition have been taken into account through the representative surface energy and molar volume of Na.^4,5^ The morphological difference between Na and Li has been shown in **Figure S7** for a representative time instant, showing that Na deposits are larger in volume and more branched, compared to Li.

Equation (2) represents the Nernst-Planck equation with a sink term $\frac{cₛ}{c^{0}}\frac{\partial\xi}{\partial t}$ accounting for the ionic consumption. Under Soret effect^6^, this equation is modified as:

$$\begin{aligned} \frac{\partial c₊}{\partial t}= \nabla\cdot[D_{eff} (\nabla c₊ + \frac{nFc₊\nabla\phi}{RT}+S_{T}c₊\nabla T)] - \frac{cₛ}{c^{0}}\frac{\partial\xi}{\partial t} \#\left( 4 \right) \end{aligned}$$

where $S_{T}=\frac{D_{T}}{D_{eff}}$ , $D_{T}$ being the thermodiffusion coefficient. A representative magnitude of Soret coefficient $\left| S_{T} \right|$ ranging between 10^-2^ to 10^-1^ has been considered in the study, which has been evidenced in literature.^7,8^ The reason behind choosing a relatively high magnitude of the Soret coefficient was to make its specific effect more prominent and to understand the mechanisms driving the electrodeposition process in the presence of thermal gradients. As may be seen from Figure 6, at S_T_ 10^-2^, Soret effect induced stabilization (or destabilization, depending on the directionality of the ions) is relatively small and its effect is not as dominant. While at S_T_ 10^-1^ the effects are more prominent. It may also be noted that even if we consider a different magnitude of the Soret coefficient, its effects will stay similar and will just change in prominence. Further, both thermophobic and thermophilic behavior of the solvated Na^+^ ions have been considered, reflected by positive and negative values of S_T_, respectively.^9^ This change of S_T_ sign (resulting in the change of thermodiffusion paradigm) depends on composition and temperature and has been represented by Bresme and Vasey.^7^ For the thermodiffusion specific results using the PFM, S_T_ representative magnitude equal to 10^-1^ has been used in the study, unless otherwise specified. Equation (3) shows the potential distribution using a Poisson type equation. The boundary and initial conditions that help define the system fully have been shown schematically in **Figure S1.**


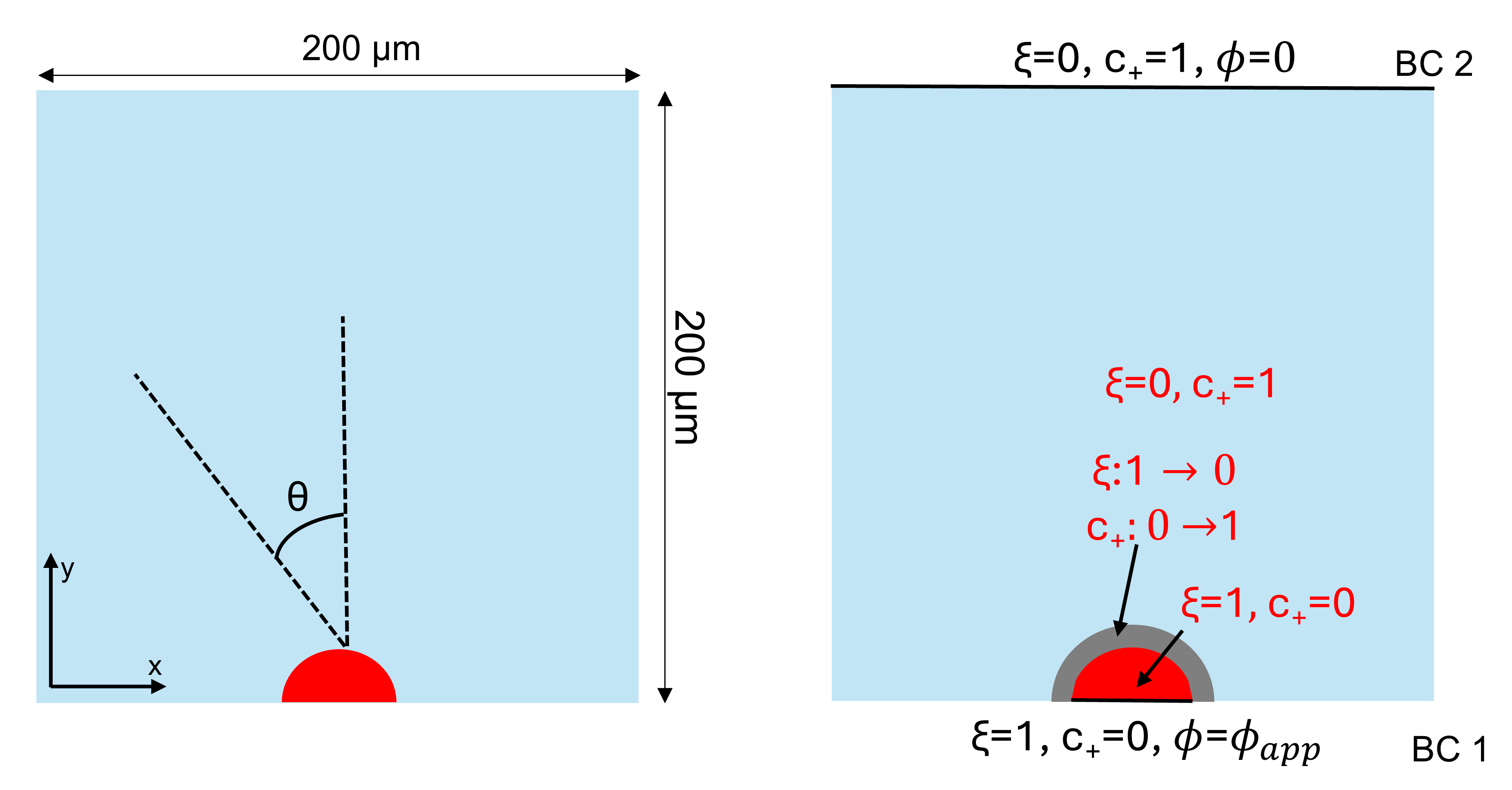


**Figure S1:** Schematic of PFM with Boundary Conditions (BCs) and Initial Conditions (ICs- in red).

**Table S1.** Parameter values for PF model

| **Parameters** | **Description** | **Value** |
| --- | --- | --- |
| $\gamma$ | Surface energy | 0.5 J m^-2^ |
| $i_{0}$ | Exchange current density | 0.5 A m^-2^ |
| $n$ | Valence | 1 |
| $\delta_{PF}$ | Phase field interface thickness | 0.5 µm |
| W | Barrier height | 1.2×10^7^ J m^-3^ |
| $k_{0}$ | Gradient energy coefficient | 3.7×10^-7^ J m^-1^ |
| $\delta$ | Anisotropy strength | 0.05 |
| $D_{s}$ | Ionic diffusivity in solid phase | 5.0×10^-15^ m^2^ s^-1^ |
| $D_{l}$ | Ionic diffusivity in electrolyte phase | 3.0×10^-10^ m^2^ s^-1^ |
| $\sigma_{s}$ | Electronic conductivity in solid phase | 2.0×10^7^ S m^-1^ |
| $\sigma_{l}$ | Electronic conductivity in electrolyte phase | 1.0 S m^-1^ |
| $cₛ$ | Molar concentration in solid phase | 4.21×10^4^ mol m^-3^ |
| $E_{a,k0}$ | Activation Energy (kinetics) | 2.0×10^4^ J mol^-1^ |
| $E_{a,d}$ | Activation Energy (diffusion) | 1.55×10^4^ J mol^-1^ |

An applied potential $\phi_{app}=-0.7V$ has been used for PFM in the present study. The rationale behind such a relatively high value derives from the derivation of Equation (1) which assumes that activation overpotential is much larger than concentration overpotential.^1^ Effective property values $D_{eff}$ and $\sigma_{eff}$ have been used in Equations (2) and (3) by interpolation using $h\left( \xi\right)$ such that:

$$\begin{aligned} D_{eff}= D_{s}h\left( \xi\right)+D_{l}\left( 1-h\left( \xi\right) \right) \#\left( 5 \right) \end{aligned}$$

$$\begin{aligned} \sigma_{eff}= \sigma_{s}h\left( \xi\right)+\sigma_{l}\left( 1-h\left( \xi\right) \right) \#\left( 6 \right) \end{aligned}$$

1. **Linear Stability Analysis (LSA) model**

In the current study, a 1D LSA model has been derived based on existing literature.^10^ The non-dimensional governing equations for the system in the bulk can be given as:

$$\begin{aligned} J_{\pm}=-D_{\pm}\left( \nabla c_{\pm}+\frac{z_{\pm}c_{\pm}F}{RT}\nabla\phi+S_{T}c_{+}\nabla T \right)\#\left( 7 \right) \end{aligned}$$

$$\begin{aligned} \frac{\partial c_{\pm}}{\partial t}=-\nabla\cdot J_{\pm}\#\left( 8 \right) \end{aligned}$$

$$\begin{aligned} -\epsilon_{P}\nabla^{2}\phi={F(z}_{+}c_{+}+z_{-}c_{-})\#\left( 9 \right) \end{aligned}$$

where, $D_{\pm}$ represents ionic diffusivity, $\epsilon_{P}$ is permittivity, $z_{\pm}$ represents valence, $S_{T}$ the Soret coefficient.


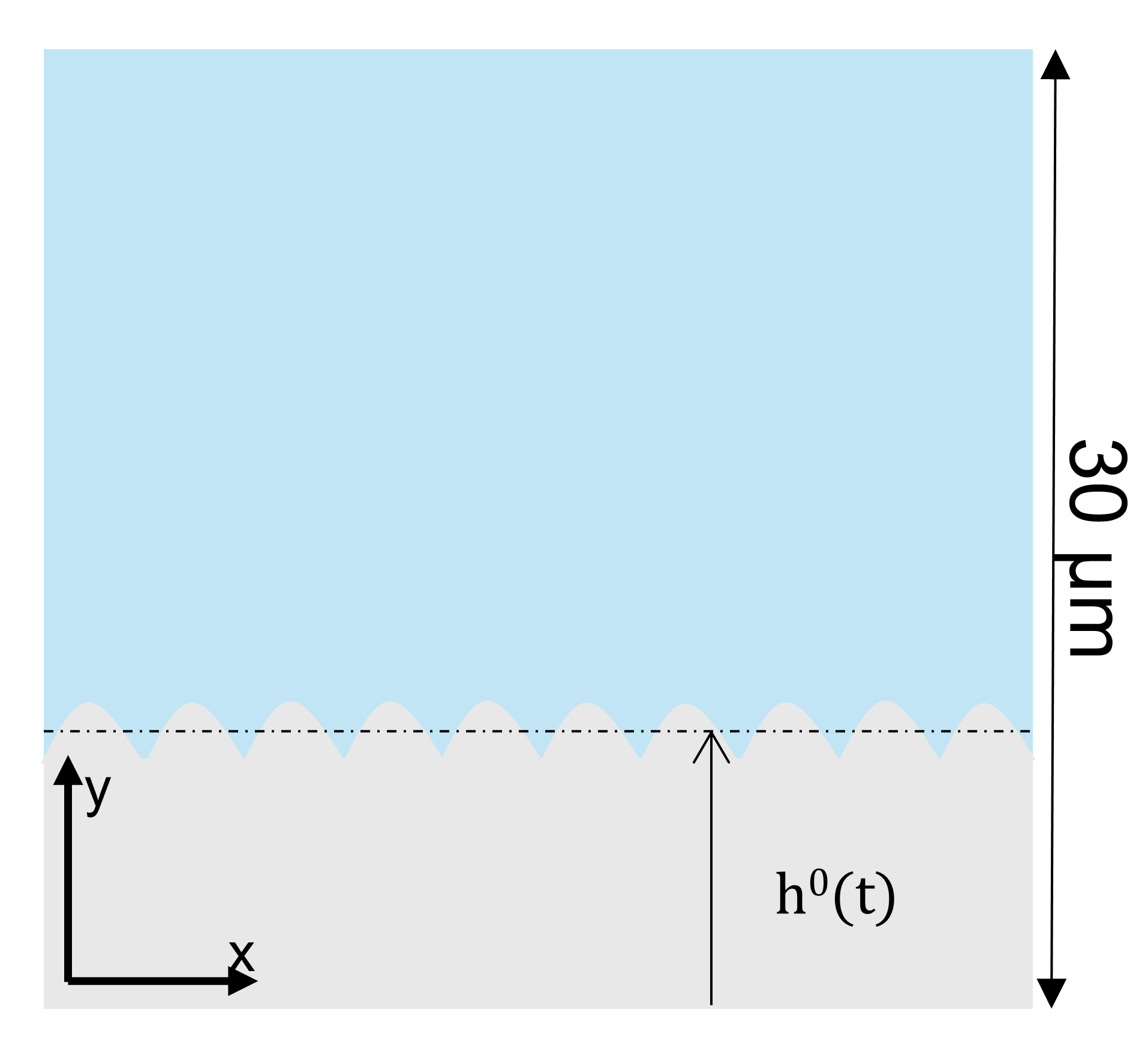


**Figure S2:** Schematic of the LSA model.

A small perturbation of the form $\epsilon e^{\omega t+ikx}$ is applied to base state variables: $h^{\left( 0 \right)}(t)$ -representing the uniform dynamic interface front under no perturbation, $c_{\pm}^{\left( 0 \right)}\left( y \right)$- the base state ionic concentration , $\phi^{\left( 0 \right)}\left( y \right)$- the base state electric potential. $\epsilon$ is the perturbation amplitude; $\omega$ is the perturbation growth rate (a higher growth rate represents greater instability) and $k$ represents the wavenumber which correlates with the perturbation morphology (a very low wavenumber would correspond to a relatively flatter interface while a higher wavenumber value would imply a rougher interface morphology).

Under the application of the perturbation, the coupled variables are modified as follows:

$$\begin{aligned} h\left( x,t \right)=h^{\left( 0 \right)}\left( t \right)+\epsilon h^{\left( 1 \right)}e^{\omega t+ikx}\#\left( 10 \right) \end{aligned}$$

$$\begin{aligned} \phi\left( x,y,t \right)=\phi^{\left( 0 \right)}\left( y \right)+\epsilon\phi^{\left( 1 \right)}\left( y \right)e^{\omega t+ikx}\#\left( 11 \right) \end{aligned}$$

$$\begin{aligned} c_{\pm}\left( x,y,t \right)=c_{\pm}^{\left( 0 \right)}\left( y \right)+\epsilon c_{\pm}^{\left( 1 \right)}\left( y \right)e^{\omega t+ikx}\#\left( 12 \right) \end{aligned}$$

Where, the 0^th^ order base state and 1^st^ order perturbed state have been marked with respective superscripts. On applying them in the main non dimensional governing equations and separating the base-state and perturbed-state terms:

$$\begin{aligned} \frac{dJ_{\pm}^{\left( 0 \right)}}{d\zeta}=0, J_{\pm}^{\left( 0 \right)}=-D_{\pm}\left( \frac{dc_{\pm}^{\left( 0 \right)}}{d\zeta}+z_{\pm}c_{\pm}^{\left( 0 \right)}\frac{d\phi^{\left( 0 \right)}}{d\zeta}+S_{T}c_{+}^{\left( 0 \right)}\frac{dT}{d\zeta} \right), -\frac{d^{2}\phi^{\left( 0 \right)}}{d\zeta^{2}}=\frac{z_{+}c_{+}^{\left( 0 \right)}+z_{-}c_{-}^{\left( 0 \right)}}{2\lambda_{D}^{2}}\#\left( 13 \right) \end{aligned}$$

$$\begin{aligned} J_{\pm}^{\left( 1 \right)}=-D_{+}\left( \frac{dc_{\pm}^{\left( 1 \right)}}{d\zeta}+z_{\pm}c_{\pm}^{\left( 1 \right)}\frac{d\phi^{\left( 0 \right)}}{d\zeta}+z_{\pm}c_{\pm}^{\left( 0 \right)}\frac{d\phi^{\left( 1 \right)}}{d\zeta}{+S}_{T}c_{+}^{\left( 1 \right)}\frac{dT}{d\zeta} \right)\#\left( 14 \right) \end{aligned}$$

$$\begin{aligned} -\frac{dJ_{\pm}^{\left( 1 \right)}}{d\zeta}=\left( \omega+D_{\pm}k^{2} \right)c_{\pm}^{\left( 1 \right)}+D_{\pm}k^{2}z_{\pm}c_{\pm}^{\left( 0 \right)}\phi^{\left( 1 \right)}\#\left( 15 \right) \end{aligned}$$

$$\begin{aligned} -\frac{d^{2}\phi^{\left( 1 \right)}}{d\zeta^{2}}+k^{2}\phi^{\left( 1 \right)}=\frac{z_{+}c_{+}^{\left( 1 \right)}+z_{-}c_{-}^{\left( 1 \right)}}{2\lambda_{D}^{2}}\#\left( 16 \right) \end{aligned}$$

where, $\zeta$ represents the moving, non-dimensional coordinate system and $\lambda_{D}^{2}=\frac{RT\epsilon_{P}}{2L^{2}F^{2}c_{0}}$

The boundary conditions at $\zeta$=0:

$$\begin{aligned} \omega h^{\left( 1 \right)}=V_{m}c_{0}R_{Li}^{\left( 1 \right)}\#\left( 17 \right) \end{aligned}$$

$$\begin{aligned} \frac{d\hat{c_{+}^{\left( 1 \right)}}}{d\zeta}=0, \hat{J_{-}^{\left( 1 \right)}}=0,-\hat{J_{+}^{\left( 1 \right)}}=R_{Li}^{\left( 1 \right)}\#\left( 18 \right) \end{aligned}$$

where, $\begin{aligned} R_{Li}^{\left( 1 \right)}=k_{0}e^{-\alpha_{\text{cat}}z\eta_{\alpha}^{\left( 0 \right)}}\left[ \frac{\hat{c_{+}^{\left( 1 \right)}}}{c_{+}^{\Theta}}+\left( \left( 1-\alpha_{\text{cat}} \right)e^{z\eta_{\alpha}^{\left( 0 \right)}}+\alpha_{\text{cat}}\frac{c_{+}^{\left( 0 \right)}}{c_{+}^{\Theta}} \right)\left( z\hat{\phi^{\left( 1 \right)}}-Cak^{2}h^{\left( 1 \right)} \right) \right]\#\left( 19 \right) \end{aligned}$

$$\begin{aligned} \hat{J_{\pm}^{\left( 1 \right)}}=-D_{\pm}\left( \frac{d\hat{c_{\pm}^{\left( 1 \right)}}}{d\zeta}+z_{\pm}c_{\pm}^{\left( 1 \right)}\frac{d\phi^{\left( 0 \right)}}{d\zeta}+z_{\pm}c_{\pm}^{\left( 0 \right)}\frac{d\hat{\phi^{\left( 1 \right)}}}{d\zeta}{+S}_{T}c_{+}^{\left( 1 \right)}\frac{dT}{d\zeta} \right)\#\left( 20 \right) \end{aligned}$$

$$\begin{aligned} \hat{\phi^{\left( 1 \right)}}=h^{\left( 1 \right)}\frac{d\phi^{\left( 0 \right)}}{d\zeta}+\phi^{\left( 1 \right)}; \hat{c_{\pm}^{\left( 1 \right)}}=h^{\left( 1 \right)}\frac{dc_{\pm}^{\left( 0 \right)}}{d\zeta}+c_{\pm}^{\left( 1 \right)}\#\left( 21 \right) \end{aligned}$$

Similarly, at $\zeta$=1:

$$\begin{aligned} \phi^{\left( 1 \right)}\left( 1 \right)=0,c_{+}^{\left( 1 \right)}\left( 1 \right)=0,c_{-}^{\left( 1 \right)}\left( 1 \right)=0\#\left( 22 \right) \end{aligned}$$

Here, $Ca=\frac{V_{m}\gamma}{RTL}$ is the non-dimensional surface energy. It may also be noted that a higher wavenumber value rapidly increases the surface energy contribution in suppressing growth and promoting stability, which derives from the fact that a higher wavenumber essentially implies more perturbed interfaces and hence a greater surface energy contribution in its resulting behavior.

**Table S2.** Parameter values for LSA model

| **Parameters** | **Description** | **Value** |
| --- | --- | --- |
| $\gamma$ | Surface energy | 0.5 J m^-2^ |
| $L$ | Domain length | 30 µm |
| $c_{0}$ | Reference Concentration | 1000 mol m^-3^ |
| $D_{l}$ | Ionic diffusivity in electrolyte phase | 3.0×10^-10^ m^2^ s^-1^ |
| $\epsilon_{P}$ | Relative Permittivity | 90 |
| $z$ | Valence | 1 |
| $cₛ$ | Molar concentration in solid phase | 4.21×10^4^ mol m^-3^ |
| $k_{0}$ | Reaction rate constant | 1.0×10^-3^ mol m^-2^ s^-1^ |
| $E_{a,k0}$ | Activation Energy (kinetics) | 2.0×10^4^ J mol^-1^ |
| $E_{a,d}$ | Activation Energy (diffusion) | 1.55×10^4^ J mol^-1^ |

For LSA analysis, an applied potential $\phi_{app,high}=-0.6V$ and $\phi_{app,low}=-0.1V$ were used. The upper limit of applied voltage was limited by convergence issues at higher $\phi_{app}$ for the property values in **Table S2**. For similar reasons, under Soret effect, all results have been shown for $\phi_{app,low}=-0.1V$. It can be noted from **Figure 6** that growth rates are negative for practically all wave numbers because of the low applied voltage, which promotes less perturbation. It might also be noted that due to the smaller interelectrode separation distance considered for LSA than in PFM, the thermal gradients are higher compared to PFM results, which leads to a dominant Soret effect and helps explain the effects of this phenomenon clearly.

1. **Additional results**

**Figure S3** schematically represents the concept of the two normalized metrics in the study: N_AA_ and N_DE_. The interfacial active area is represented by the gray boundary at the deposit boundary while the depleted electrolyte zone is highlighted in blue.


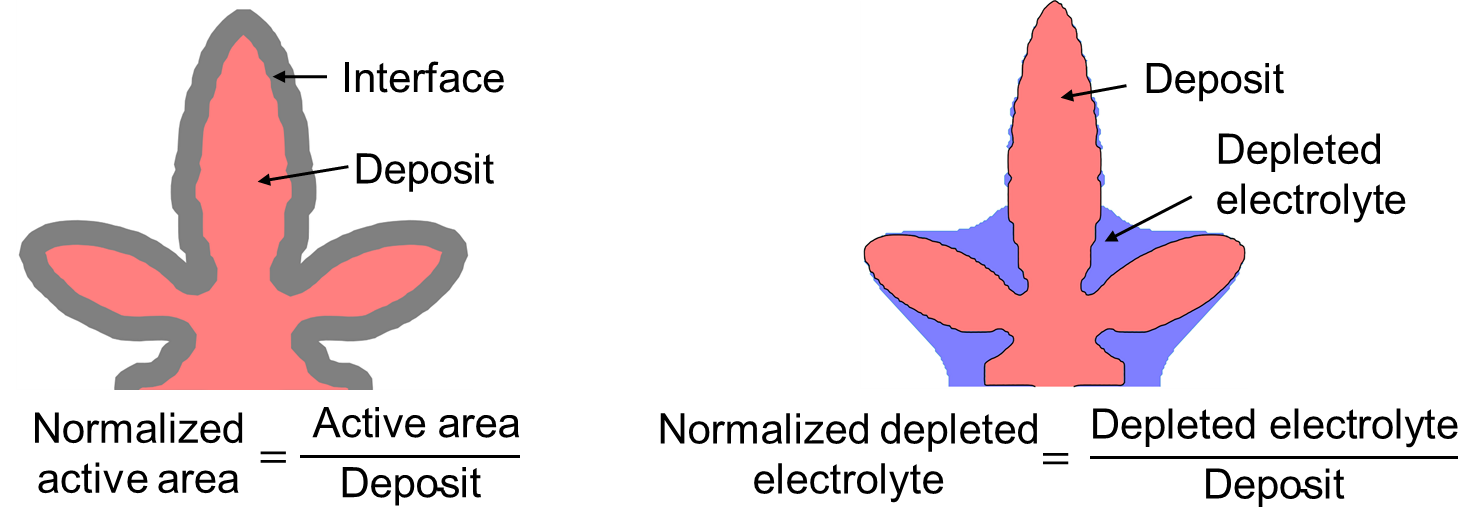


**Figure S3:** Schematic representation of normalized active area (N_AA_) and normalized depleted electrolyte (N_DE_).

**Figure S4** shows a phase map of the two normalized metrics of instability defined in the study- the normalized active area (N_AA_) and normalized depleted electrolyte (N_DE_) at t_1_ and t_2_ times. While correlated, N_DE_ phase map shows a more consistent distribution of the stable and unstable zones and less fluctuating behavior when compared to N_AA._ This is likely due to the larger zone covered by the depleted electrolyte occupying regions adjacent to the deposit, while the active area is confined only to the interface- hence is subject to small numerical deviations. Thus, in the present study, while analyzing instability under various operational regimes, N_DE_ has been preferably shown.

**
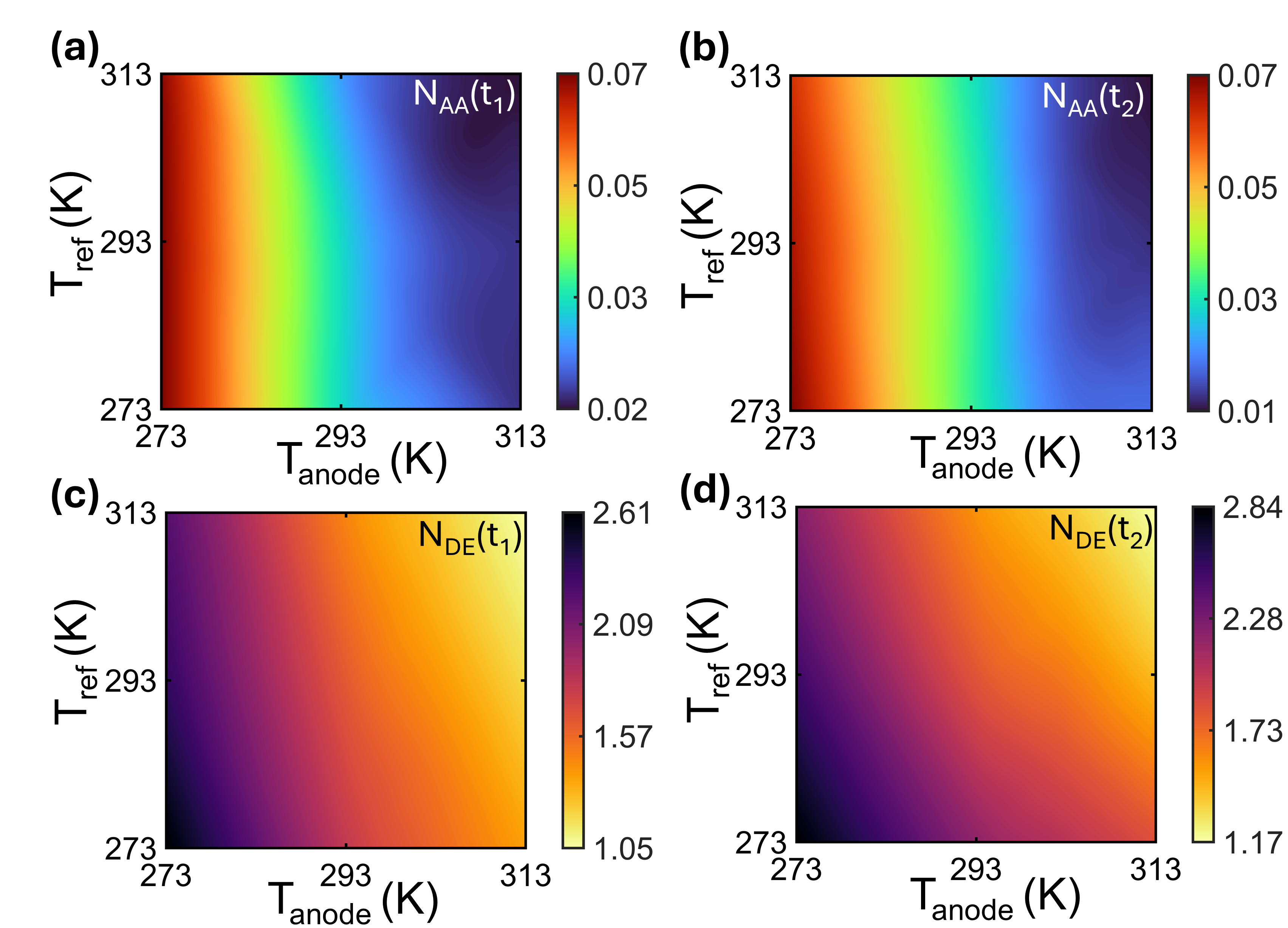
**

**Figure S4:** Phase map of stability with varying anode and reference temperatures shows a comparison of the consistency between N_AA_ (top row) and N_DE_ (bottom row) at two times t_1_ and t_2_ (t_2_>t_1_).

**Figure S5** shows LSA analysis for uniform and differential temperature conditions at high and low applied plating potential values (ɸ_e_= -0.6V, -0.1V). For low applied potential (ɸ_e_= -0.1V), ω is monotonically negative implying that all surface perturbations eventually vanish irrespective of the nature of the perturbation applied (k value). For higher magnitude of negative applied potential, positive ω values are observed reaching a maximum value (ω_max_) at critical wavenumber (k_cr_). For both thermal conditions shown, it may be observed that low uniform temperature, and specifically low anode temperature conditions promote surface instability- the corresponding ω values being higher for these scenarios for all k values. This corresponds with the results from PFM.

~~
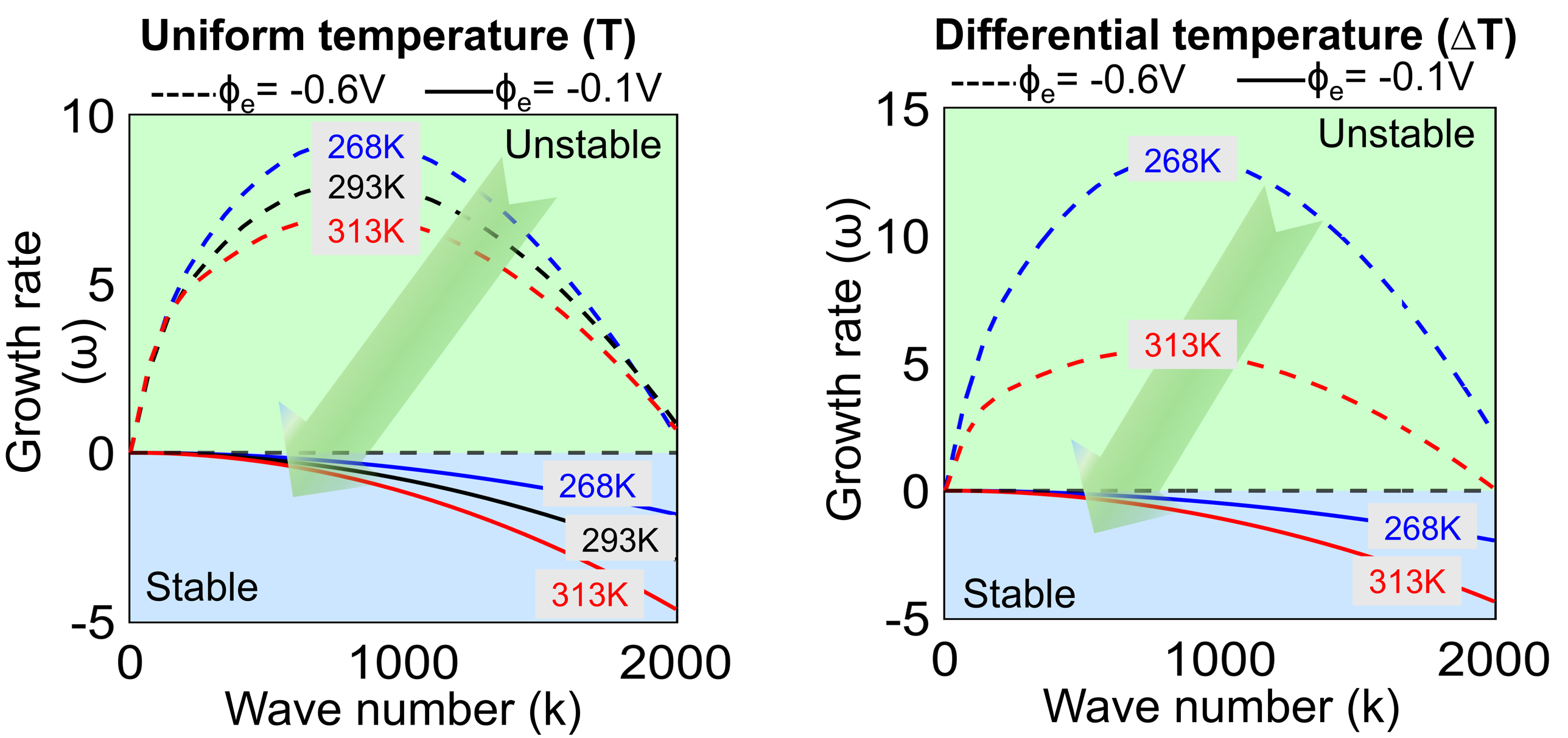
~~

**Figure S5:** LSA comparison of surface perturbation under uniform and differential temperature conditions.

The importance of normalization in defining the instability metrics is shown in **Figure S6**, corresponding to the thermodiffusion conditions shown in **Figure 5** (**Section 2.3**). While N_DE_ in **Figure 5** shows a clear trend of instability, plots of *total depleted electrolyte* (**Figure S6**) largely overlap and so no such clear demarcation under the different thermodiffusion conditions, showing that it is crucial to use normalized metrics when comparing relative instability.


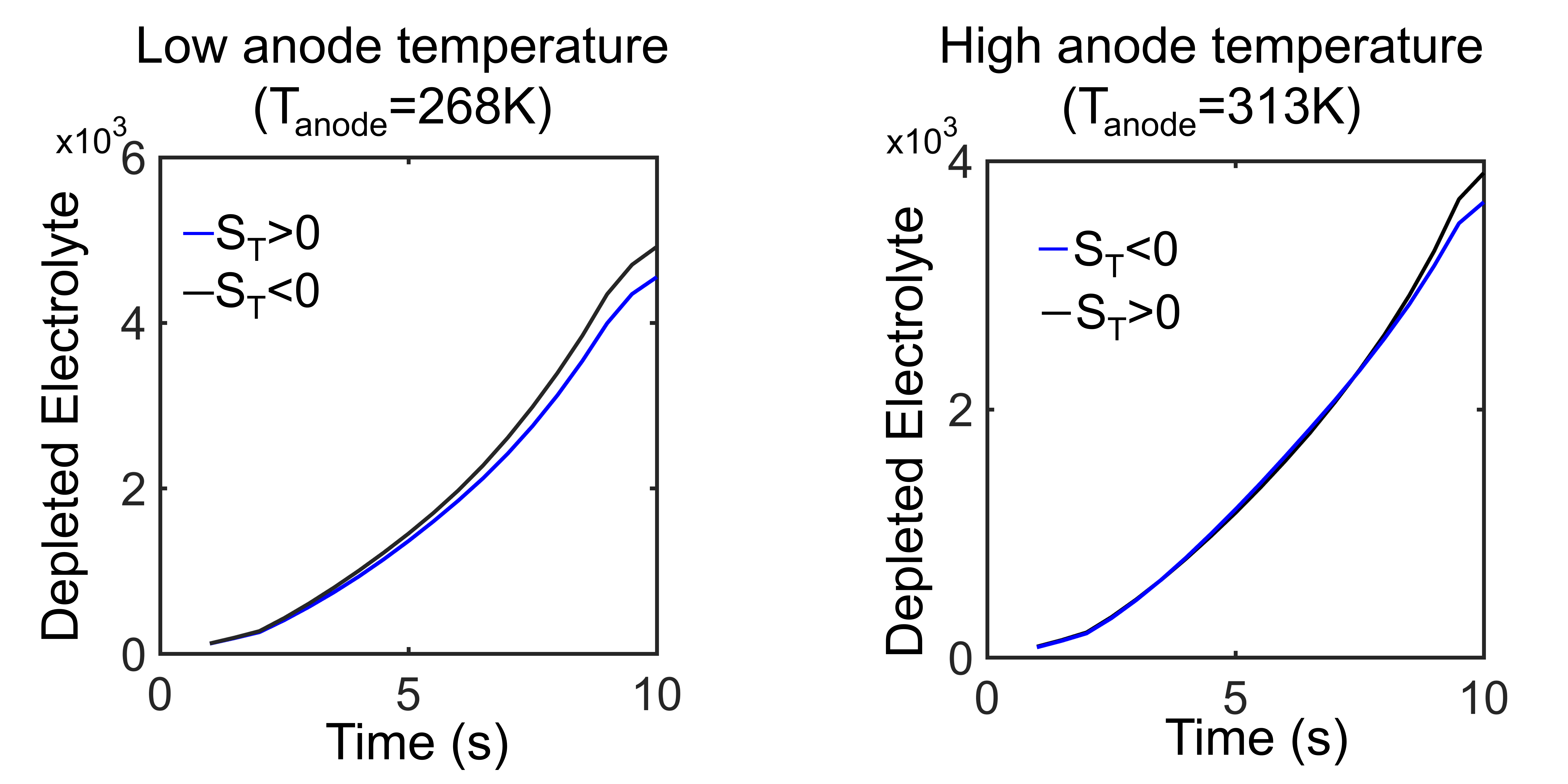


**Figure S6:** Total depleted electrolyte variation with time. It may be observed that the line plots largely overlap with no clear trend.

Lastly, a comparison between Na and Li electrodeposits has been shown in **Figure S7**, where it can be seen that the Na electrodeposit is larger, and more dendritic compared to Li for the same time instant. This morphologically depicts the manifestations of a higher molar volume and lower surface energy for Na as compared to Li.


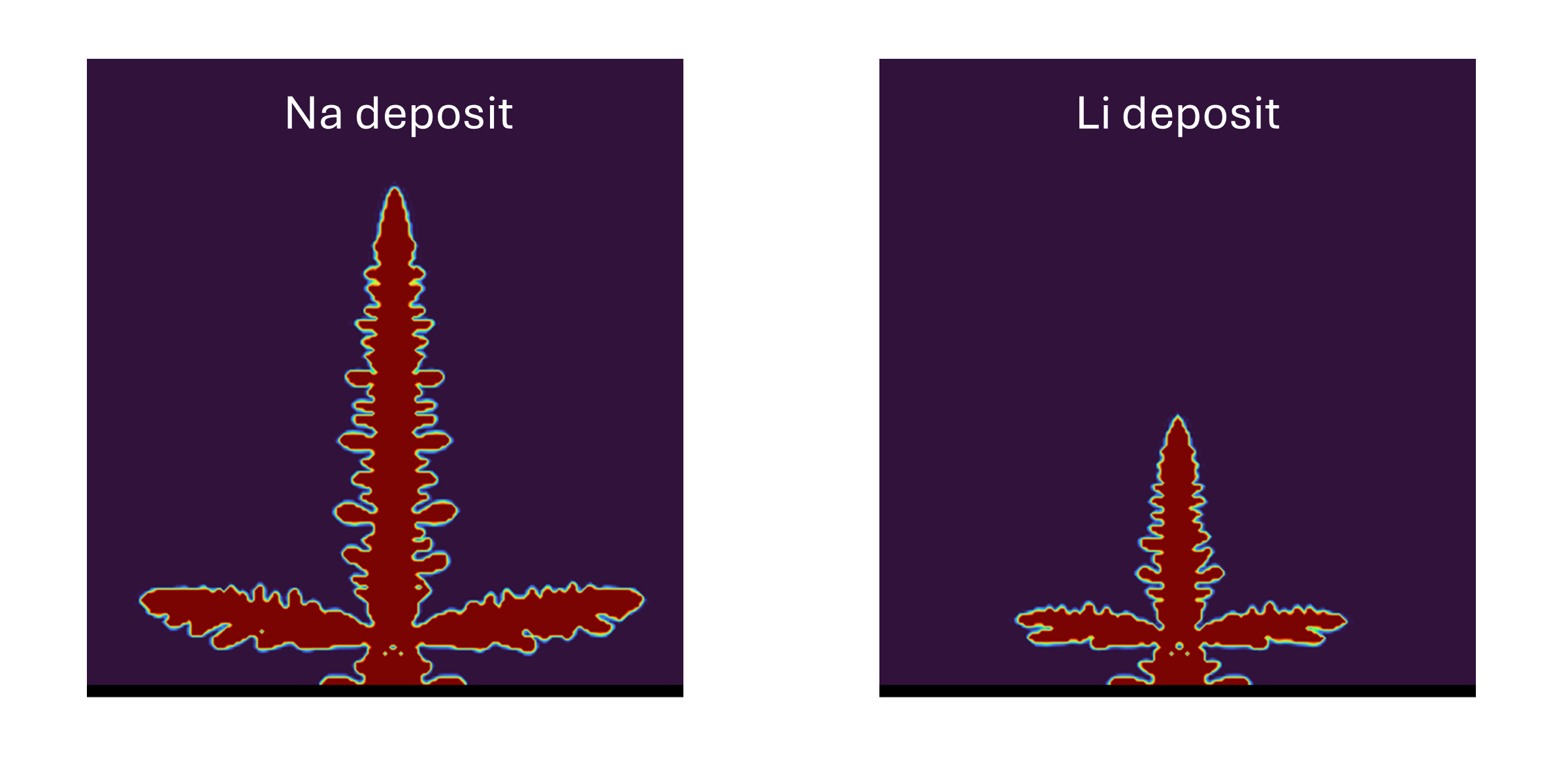


**Figure S7:** Morphological comparison between Na and Li electrodeposit at the same time instant.

**References**

1. Chen, L. *et al.* Modulation of dendritic patterns during electrodeposition: A nonlinear phase-field model. *Journal of Power Sources* **300**, 376–385 (2015).

2. Arguello, M. E., Gumulya, M., Derksen, J., Utikar, R. & Calo, V. M. Phase-field modeling of planar interface electrodeposition in lithium-metal batteries. *Journal of Energy Storage* **50**, 104627 (2022).

3. Xiong, Y., Yan, B., Li, Q., Zhi, C. & Fan, J. Phase field modeling of dendrite growth mechanism of Mg and Li in electrodeposition. *Journal of Power Sources* **597**, 234162 (2024).

4. Vitos, L., Ruban, A. V., Skriver, H. L. & Kollár, J. The surface energy of metals. *Surface Science* **411**, 186–202 (1998).

5. Lee, B., Paek, E., Mitlin, D. & Lee, S. W. Sodium Metal Anodes: Emerging Solutions to Dendrite Growth. *Chem. Rev.* **119**, 5416–5460 (2019).

6. Zhao, D., Würger, A. & Crispin, X. Ionic thermoelectric materials and devices. *Journal of Energy Chemistry* **61**, 88–103 (2021).

7. Bresme, F. & Vasey, E. Thermal transport of alkali halide aqueous solutions: a non-equilibrium molecular dynamics investigation. *Molecular Physics* **122**, e2388302 (2024).

8. Rosanne, R., Paszkuta, M., Tevissen, E. & Adler, P. M. Thermodiffusion in compact clays. *Journal of Colloid and Interface Science* **267**, 194–203 (2003).

9. Mohanakumar, S. & Wiegand, S. Towards understanding specific ion effects in aqueous media using thermodiffusion. *Eur. Phys. J. E* **45**, 10 (2022).

10. Li, W., Tchelepi, H. A., Ju, Y. & Tartakovsky, D. M. Stability-Guided Strategies to Mitigate Dendritic Growth in Lithium-Metal Batteries. *J. Electrochem. Soc.* **169**, 060536 (2022).
